# Family caregivers’ support needs during allo-HSCT—a longitudinal study

**DOI:** 10.1007/s00520-020-05853-8

**Published:** 2020-10-30

**Authors:** Annika M. Kisch, Karin Bergkvist, Anette Alvariza, Kristofer Årestedt, Jeanette Winterling

**Affiliations:** 1 Haematology Department, Lund University Hospital, Lund, Sweden; 2 Institute of Health Sciences, Lund University, Lund, Sweden; 3 Department of Neurobiology, Care Sciences and Society, Karolinska Institutet, Stockholm, Sweden; 4 Sophiahemmet University, Stockholm, Sweden; 5 Department of Health Care Sciences/Palliative Research Centre, Ersta Sköndal Bräcke University College, Stockholm, Sweden; 6 Faculty of Health and Life Sciences, Linnaeus University, Kalmar, Sweden; 7 The Research Section, Region Kalmar County, Kalmar, Sweden; 8 Medical Unit Hematology, Theme Cancer, Karolinska University Hospital, Stockholm, Sweden

**Keywords:** Allo-HSCT, CSNAT, Family caregivers, Haematological malignancies, Support needs

## Abstract

**Purpose:**

The study aimed to explore family caregivers’ support needs prior to allo-HSCT, how these change over time and whether they are associated with demographic factors and caregiver outcome.

**Methods:**

This longitudinal repeated measure study included 87 family caregivers of allo-HSCT recipients: 63% were partners, 74% women, 65% lived with the recipient, and their mean age was 54 years. They completed the 14-item Carer Support Needs Assessment Tool (CSNAT) and caregiver outcome measures (caregiver burden, anxiety, depression, preparedness for caregiving and general health) prior to allo-HSCT and 3, 6 and 16 weeks later.

**Results:**

The two top support needs prior to allo-HSCT were ‘knowing what to expect in the future’ (79%) and ‘dealing with your own feelings’ (70%). Several support needs were associated with younger age and not being a partner, while higher needs implied worse caregiver outcomes for at least one of the outcomes prior to transplantation. Most support needs remained the same at the last follow-up.

**Conclusion:**

The findings that high support needs are often associated with worse caregiver outcomes and most support needs do not diminish over time indicate that more attention should be placed on the situation of family caregivers.

## Introduction

Allogeneic hematopoietic stem cell transplantation (allo-HSCT) is a treatment mainly for haematological malignancies. In Europe, around 17,000 allo-HSCTs are performed per year and in Sweden around 280 [1]. The goal with the treatment is to cure the patient; however, the treatment is very demanding with numerous side effects, long period of in-patient care and risks for complications and includes a long recovery period [2]. Most recipients of allogeneic hematopoietic stem cell transplantation (allo-HSCT) need help with the activities of daily life during the transplantation trajectory, and family members more or less willingly become caregivers. Being responsible for physical as well as emotional care can be challenging for family caregivers (FC) [3] who need to cope with their own stress and worries about the future [4, 5]. FC often show high levels of distress, sometimes even higher than the recipients [4]. Until now, research on FC of HSCT recipients has mainly focused on their experiences [6–8], quality of life [6], psychological distress [7, 9–11] and caregiver burden [7, 12].

It is important to explore FC support needs during allo-HSCT. However, few studies have focused on this aspect, as only two qualitative studies exclusively on allo-HSCT [13, 14] and three quantitative studies on allo- and auto-HSCT were identified. These studies indicate that FC have unmet information, psychological and social needs [14–17]. One of the studies explored the relationship between support needs and other caregiver outcomes, indicating that higher levels of distress and lower levels of general health are associated with higher support needs [15]. Similar relationships are reported in studies on palliative cancer care [18, 19], and the relationship between higher levels of distress and higher support needs is also found within curative cancer care [20–23]. It has been acknowledged that feelings of being prepared for caregiving and caregiver burden are associated with less need of support in palliative care [18, 19]. The association between sociodemographic data and FC support needs is not well known, and existing results are somewhat contradictory [20–22]. Earlier studies have described that the most common FC of allo-HSCT patients are spouses/partners, but also other relatives or friends, and the majority are female [4]. Apart from our recent qualitative study [13], only one other study focusing exclusively on support needs of FC of allo-HSCT recipients was identified, which is a qualitative study solely exploring information needs and performed more than two decades ago [14]. Since then, many circumstances have changed, e.g. the preparative regimen, the care procedure and the nursing actions [24, 25]. To conclude, the knowledge about FC support needs from before allo-HSCT and during the acute post-transplantation phase is scarce as we could only find one longitudinal study on this topic [17]. The aim of this study was to explore FC support needs prior to allo-HSCT, how these change over time and whether they are associated with demographic factors and caregiver outcome. Here, time refers to from before until 4 months after allo-HSCT.

## Method

### Design

The study had a longitudinal repeated measure design. Questionnaires were used prior to the start of the allo-HSCT treatment (baseline) and at follow-up at 3, 6 and 16 weeks after transplantation. The three time points after allo-HSCT were chosen with the purpose to capture the FC support needs during the acute post-transplantation phase, when the situation for the patient is quite intense, and so also probably for the FC. Three weeks after allo-HSCT, the patient is usually still at the hospital but on the way to be discharged, and many questions and worries may arise for the FC. Six weeks after the transplantation, the patient has just settled at home with new areas for concerns, and 16 weeks after allo-HSCT, the patient is usually back to a kind of a new everyday life, which may imply a new situation for the FC including new issues and needs.

### Sample

**Fig. 1:**
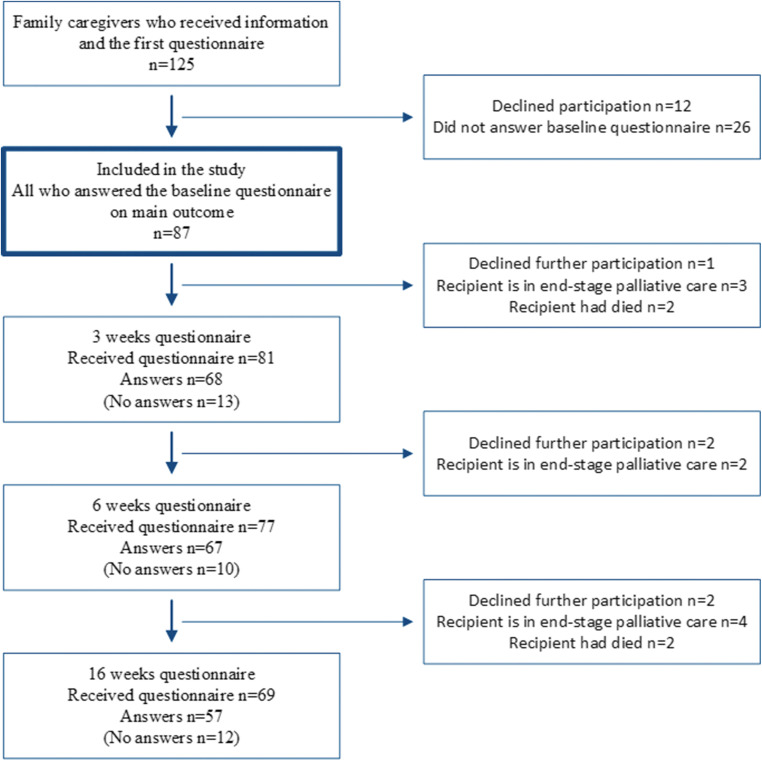
The participation and attrition rate over time

The inclusion criteria were adult (≥ 18 years) FC able to read and write Swedish and selected by an allo-HSCT recipient transplanted at two of the six transplantation centres in Sweden. During the inclusion period from October 15, 2017, to November 14, 2018, 148 recipients were transplanted, of whom three stated that they had no FC and three did not want their FC to be asked; thus, 142 recipients selected an FC. Of these 17 FC were excluded due to not understanding Swedish. Among the 125 eligible FC, 12 did not want to participate in the study, and 26 failed to answer the baseline questionnaire, resulting in a study cohort of 87 FC (response rate 87/125 = 70%). The participation and attrition rate over time is presented in Fig. 1.

### Procedure and measurements

Recipients were asked by the HSCT coordinator either by phone or in person to select an FC involved in everyday life and circumstances around the disease. If the recipient agreed, the HSCT coordinator sent the study information letter and baseline self-administered questionnaire to the selected FC and informed the study coordinator. In some cases, the FC attended the meeting with the recipient and received oral and written information. The study coordinator sent the follow-up questionnaires to the FC, and if they were not returned, the FC were reminded twice by phone. For the follow-up assessments, questionnaires were sent to all FC unless they had declined further participation, or if the recipient had died or was close to death. The study coordinator was continuously informed by the HSCT coordinators if the health status of a patient had deteriorated significantly. The questionnaires included the following self-report instruments.

The Carer Support Needs Assessment Tool (CSNAT) is the main outcome measure and an evidence-based comprehensive practice tool for measuring domains of FC support needs in palliative care [26]. It consists of 14 items with 4 response alternatives for each item: “no more (1),” “a little more (2),” “quite a bit more (3)” or “very much more (4)”. It covers enabling (7 items) and direct support (7 items) needs (Box 1). The Swedish version of the CSNAT has shown satisfactory validity and reliability [18].

Box 1 Support domains of the Carer Support Needs Assessment Tool (CSNAT).

**Table:** 

	The carer identifies whether he/she needs more support with the following domains
Enabling support needs - Seven domains of support enabling the family caregiver to care for the patient	• Understanding their relative’s illness • Managing their relative’s symptoms, including giving medicines • Providing personal care (e.g. dressing, washing, toileting) • Knowing whom to contact when concerned • Equipment to help care for their relative • Talking with their relative about his/her illness • Knowing what to expect in the future when caring for their relative
Direct support needs - Seven domains of support in relation to family caregiver’s own well-being	• Looking after his/her own physical health • Having time for oneself in the day • Any financial, legal or work issues • Dealing with feelings and worries • Beliefs or spiritual concerns • Practical help in the home • Getting a break from caring overnight

The Caregiver Burden Scale (CBS) measures subjective burden experienced by caregivers [27]. It consists of 22 items, all rated on a 4-point Likert-type scale, ranging from 1 to 4. In the present study, only one of the five sub-scales was used: the “general strain” scale (CBS-GS). The mean ratings on the eight CBS-GS items are calculated with a possible score ranging from 1 to 4, where a higher score indicates higher caregiver burden. The scale has shown satisfactory validity and reliability [27].

The Hospital Anxiety and Depression Scale (HADS) consists of two sub-scales with 7 items each, one measuring symptoms of anxiety and the other depression [28]. Each item has four response alternatives, ranging from 0 to 3. The sub-scale score ranges from 0 to 21, with a higher score indicating more severe symptoms of anxiety or depression. The cut-off value is a score of ≥ 8. The HADS has shown satisfactory validity and reliability [29], also in a Swedish context [30].

The Preparedness for Caregiving Scale (PCS) measures caregivers’ readiness to provide care [31]. The scale has eight items, all rated on a 5-point Likert-type scale, ranging from 0 to 4. The total score ranges from 0 to 32, with a higher score indicating greater preparedness [31]. The scale has shown satisfactory validity and reliability [32], also the Swedish version [33].

General health was measured using one item from the Short Form 36 (SF-36) [34], “How would you rate your general health?”, which has five response alternatives ranging from “excellent” (1) to “poor” (5).

### Statistical analysis

Missing data in the CBS-GS, HADS and PCS were replaced using person mean imputation [35], if they did not exceed 20% for each scale [36]. Descriptive statistics were used to describe the characteristics of the participants and FC support needs prior to allo-HSCT.

Nonparametric tests were employed because CSNAT responses were treated as ordinal data. Spearman rank order correlation (r_s_) was used to explore whether support needs at baseline were associated with demographic factors and caregiver outcome. Since some of the items in the CSNAT have conceptual overlaps with both CBS-GS and PCS, the variance inflation factor (VIF), a measure of multicollinearity, was calculated for each CSNAT item and these two scales. As no problem with multicollinearity (VIF > 2.0) was detected for the CSNAT items and CBS-GS (VIF = 1.00 − 1.21) or PCS (VIF = 1.00 − 1.21), the correlation analyses between all CSNAT items and these two scales were supported. The Friedman test was applied to investigate whether support needs changed over time from baseline to 16 weeks later. Only participants who filled in the support needs assessment tool on all four measurement occasions were included in the analysis (*n* = 50). The Wilcoxon signed rank test was used as a post hoc test. The significance level was set at *p* < 0.05. The statistical calculations were performed using the SPSS Statistics, version 24.0 (IBM Corp., Armonk, NY, USA).

### Ethical considerations

We have considered that participating in the study and answering questionnaires may be associated with strong emotions and add an extra burden. However, it might be appreciated that the own situation of the FC is given attention. The study information emphasized the voluntary nature and the right to withdraw at any time point, further that data is treated with confidentiality and that the identity of participants is protected. The study has been approved by the Regional Ethical Review Board in Stockholm (Dnr 2017/1112-31/4).

## Results

Detailed information about the 87 FC who participated in the present study is provided in Table 1. Their mean age was 54.9 (SD = 13.2) years, and the majority were women (*n* = 66, 76%) and partners (*n* = 57, 66%).

**Table 1:** Characteristics of the participants (*n* = 87)

	*N* (%)
Age, years, mean	54.9 (13.2)
Gender, *n* (%)	
Female	66 (76)
Male	21 (24)
Education, *n* (%)	
Lower (elementary or secondary school)	40 (46**)**
Higher (college/university)	46 (53)
Missing	1 (1)
Country of birth, *n* (%)	
Sweden	79 (91)
Elsewhere	8 (9)
Relationship to recipient, *n* (%)	
Partners	57 (66)
Children	16 (18)
Parents	9 (10)
Others	5 (6)
Cohabitant with the recipient, *n* (%)	
Yes	58 (67)
No	29 (33)
Married, *n* (%)	
Yes	64 (74)
No	23 (26)
Have own healthcare issue^1^ (*n* = 87)	
Yes^2^	34 (439
No	52 (60)
Missing	1 (1)
Occupation status	
Working (full-time)	41 (47)
Working (part-time)	13 (15)
On sick-leave/disability pension	7 (8)
On old age pension	24 (27)
Other (seeking work, parental leave)	2 (3)
Children < 18 years, *n* (%)	
Yes	22 (25)
No	65 (75)

^1^Self reported data about their own healthcare issues diagnosed by a medical doctor

^2^Of these 6 reported stress or crisis reaction or depression

### Reported support needs

**Fig. 2:**
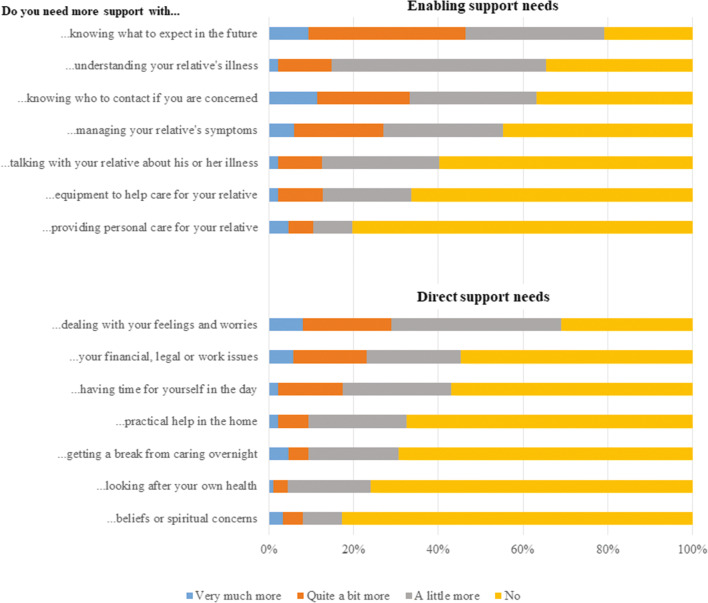
Description of family caregivers’ support needs prior to allo-HSCT (CSNAT items)

Prior to allo-HSCT, the three top support needs enabling the FC to care for the recipients, i.e. enabling support needs, were as follows: ‘knowing what to expect in the future’ (79%), ‘understanding your relative’s illness’ (66%) and ‘knowing who to contact if you are concerned’ (63%). Furthermore, the three top support needs in relation to FC well-being, i.e. direct support needs, at baseline were as follows; ‘dealing with your own feelings and worries’ (70%), ‘your financial, legal or work issues’ (45%) and ‘having time to yourself in the day’ (43%) (Fig. 2).

### Support needs and demographic factors

Of the 14 FC support needs included in the CSNAT, six were statistically significantly associated with younger age (r_s_ = − 0.22 to − 0.24) and five with not being a partner (r_s_ = 0.27 to 0.39). There were no significant associations between support needs and gender or level of education (Table 2).

**Table 2:** Associations between support needs prior to allo-HSCT, demographic data and caregiver outcome (*n* = 84–87)

	Demographic data				Caregiver outcome				
Support needs^1^	Age	Gender (male)	Education (high)	Relation (not being a partner)	Caregiver burden	Anxiety	Depression	Preparedness	General health
Enabling support needs									
Knowing what to expect in the future	− 0.22*	− 0.18	0.10	0.16	0.41***	0.32*	0.30**	− 0.28**	0.17
Understanding your relative's illness	− 0.13	− 0.07	− 0.21	0.20	0.10	0.18	0.11	− 0.38***	0.11
Knowing who to contact if concerned	− 0.24*	− 0.01	− 0.11	0.31**	0.17	0.26*	0.14	− 0.21	0.00
Managing your relative's symptoms	− 0.22*	0.07	− 0.14	0.39***	0.25*	0.24*	0.18	− 0.33**	0.14
Talking with your relative about their illness	− 0.20	0.06	− 0.08	0.11	0.24*	0.09	0.24*	− 0.36**	0.09
Equipment to help care for your relative	− 0.23*	− 0.10	− 0.17	0.27*	0.07	0.27*	0.10	− 0.27*	0.08
Providing personal care for your relative	− 0.11	0.19	− 0.18	0.28**	0.01	0.08	0.11	− 0.12	0.16
Direct support needs									
Dealing with your feelings and worries	− 0.17	− 0.07	− 0.03	0.33**	0.31**	0.43***	0.31**	− 0.38***	0.06
Your financial, legal or work issues	− 0.22*	0.06	− 0.01	0.20	0.28*	0.14	0.26*	− 0.09	0.18
Having time to yourself in the day	− 0.15	− 0.09	− 0.09	0.06	0.26*	0.13	0.28	− 0.02	0.38***
Practical help in the home	− 0.22*	0.18	− 0.01	0.05	0.22*	0.14	0.20	− 0.13	0.39***
Getting a break from caring overnight	− 0.17	0.03	− 0.16	0.18	0.25*	0.04	0.04	− 0.07	0.12
Looking after your own health	− 0.15	− 0.04	0.00	− 0.03	0.31**	0.42***	0.47***	− 0.12	0.20
Beliefs and spiritual concerns	− 0.07	− 0.12	− 0.08	0.06	0.28**	0.28**	0.26*	− 0.05	− 0.01

^1^A higher score means having higher support needs; Spearman rank order correlations, *p < 0.05, **p < 0.01, ***p < 0.001

### Support needs and caregiver outcome

The association between support needs and FC outcomes are presented in Table 2. All of the 14 support needs measured prior to allo-HSCT were significantly associated with at least one of the outcomes, i.e. higher needs implied worse outcomes. Higher support needs were significantly associated with higher levels of caregiver burden (10 out of 14, r_s_ = 0.22 to 0.41), symptoms of anxiety (7 out of 14, r_s_ = 0.24 to 0.43) and depression (6 out of 14, r_s_ = 0.24 to 0.47). In addition, higher support needs were significantly associated with lower levels of preparedness for caregiving (6 out of 14, r_s_ = − 0.27 to − 0.38) and general health (2 out of 14, r_s_ = 0.38 to 0.39).

### Changes in support needs over time

Table 3 shows changes in FC support needs over time, from prior to allo-HSCT until 4 months afterwards. A majority of the support needs, 9 out of 14, did not change over the 4-month follow-up period. However, five support needs decreased significantly over time. ‘Knowing who to contact if you are concerned’ (*p* < 0.001) and ‘equipment to help care for your relative’ (*p* < 0.001) decreased between all measurements, while ‘getting a break from caring overnight’ (*p* = 0.032) decreased from baseline to 6 weeks as well as from baseline to 16 weeks. Two support needs decreased from baseline to 16 weeks, ‘providing personal care for your relative’ (*p* = 0.008) and ‘your financial, legal or work issues’ (*p* = 0.003).

**Table 3:** Changes in caregivers’ support needs over time from baseline to 16 weeks later (*n* = 49–50)

Support needs	Baseline	3 weeks	6 weeks	16 weeks	*p* Value	Post hoc test^3^
	Md (Q1, Q4) mean rank^2^	Md (Q1, Q4) mean rank^2^	Md (Q1, Q4) mean rank^2^	Md (Q1, Q4) mean rank^2^		
Enabling support needs^1^						
Knowing what to expect in the future	2 (2, 3) 2.77	2 (2, 3) 2.49	2 (1, 3) 2.34	2 (1, 3) 2.41	0.124	-
Understanding your relative's illness	2 (1, 2) 2.48	2 (1, 2) 2.57	2 (1, 2) 2.48	2 (1, 2) 2.47	0.939	-
Knowing who to contact if concerned	2 (1, 3) 2.97	1 (1, 2) 2.46	1 (1, 2) 2.46	1 (1, 2) 2.31	0.000	a, b, c
Managing your relative's symptoms	1 (1, 3) 2.68	1 (1, 2) 2.56	1 (1, 2) 2.40	1 (1, 2) 2.36	0.167	-
Talking with your relative about their illness	1 (1, 2) 2.62	1 (1, 2) 2.54	1 (1, 2) 2.48	1 (1, 2) 2.36	0.417	-
Equipment to help care for your relative	1 (1, 2) 2.81	1 (1, 1) 2.43	1 (1, 1) 2.42	1 (1, 1) 2.34	0.000	a, b, c
Providing personal care for your relative	1 (1, 1) 2.62	1 (1, 1) 2.61	1 (1, 1) 2.46	1 (1, 1) 2.31	0.008	c
Direct support needs^1^						
Dealing with your feelings and worries	2 (1, 3) 2.65	2 (1, 2) 2.61	2 (1, 2) 2.44	2 (1, 2) 2.30	0.191	-
Your financial, legal or work issues	1 (1, 2) 2.73	1 (1, 2) 2.66	1 (1, 2) 2.47	1 (1, 2) 2.14	0.003	c
Having time to yourself in the day	1 (1, 2) 2.32	1 (1, 2) 2.54	1 (1, 2) 2.67	1 (1, 2) 2.47	0.152	-
Practical help in the home	1 (1, 2) 2.66	1 (1, 1) 2.53	1 (1, 1) 2.49	1 (1, 1) 2.32	0.113	-
Getting a break from caring overnight	1 (1, 1) 2.71	1 (1, 1) 2.52	1 (1, 1) 2.43	1 (1, 1) 2.34	0.032	b, c
Looking after your own health	1 (1, 1) 2.38	1 (1, 2) 2.60	1 (1, 1) 2.48	1 (1, 1) 2.54	0.463	-
Beliefs and spiritual concerns	1 (1, 1) 2.55	1 (1, 2) 2.57	1 (1, 1) 2.45	1 (1, 1) 2.43	0.682	-

^1^A higher score means having higher support needs; ^2^Mean ranks indicate how the groups differed, a lower mean rank implies lower support needs; ^3^Post hoc tests with Wilcoxon signed rank test between a = baseline − 3 weeks, b = baseline − 6 weeks, c = baseline − 16 weeks

## Discussion

The result shows that FC reported several support needs. The most salient result is that almost 80% needed more support with knowing what to expect in the future and 70% with how to deal with their own feelings and worries. Higher support needs were associated with worse caregiver outcomes and with younger age and not being a partner. Most support needs did not change over time.

The CSNAT was developed to cover the dual role of FC, i.e. caring for the recipient (enabling support) and coping with their own well-being (direct support).

Our results indicate that both roles need attention among FC of patients undergoing allo-HSCT. The two top support needs in each of these roles reported in our study are in line with previous studies using the CSNAT in palliative care [18, 19, 37, 38]. However, it should be highlighted that the percentage reporting these support needs prior to transplantation is higher in our study compared to those in palliative care, 79% vs. 43–68%, respectively, for ‘knowing what to expect in the future’ and 70% vs. 27–65%, respectively, for ‘dealing with your own feelings and worries’. These two top support needs that did not diminish over time are probably related to the strong sense of uncertainty in allo-HSCT, due to the high risk of relapse and the fact that the recipient’s health status often changes rapidly [5, 9]. This is confirmed by quantitative studies in the HSCT context, where the top support need was managing concerns about the cancer returning [15, 17] and the need for support to cope with fear [16]. In qualitative studies, participants also express a great need to obtain information about the recipient’s illness, treatment and future [13, 14]. As many as 63% of the FC in the present study expressed a need to know who to contact in the healthcare system if they were concerned. This was somewhat surprising, as healthcare professionals often assume that recipients and their FC are well-informed prior to transplantation. However, other studies confirm that there is a need for FC to be able to communicate with and to be heard by healthcare professionals and that this need is often unmet [13, 14].

In present study, it was mainly support needs concerning information and emotional well-being that did not change over time, while more practical support needs decreased over time. This may be because FC received information or learned along the transplant trajectory. There is a lack of longitudinal studies investigating support needs among allo-HSCT FC. However, a few longitudinal studies in cancer care show different results; among FC of patients with incurable brain cancer, support needs did not decline over a 6-month period [39], while for FC of patients with incurable ovarian cancer, support needs decreased during the last year of life [40]. FC of patients with mixed cancer diagnoses reported a decrease in unmet needs from 6 to 24 months post-diagnosis [41]. Our result reveals that many support needs do not diminish by themselves, which is in line with earlier studies showing that FC of allo-HSCT patients experience high levels of distress for a long time [10, 11, 15], which is related to the uncertainty of the situation.

The present study reveals that both being younger and not being a partner was associated with several support needs. The younger FC were mainly adult children of the patient and therefore did not live together with the patient, and thereby they might not have been as much involved in the daily living as FC living together with the patient. This indicates that those FC less involved in the patient’s care would probably benefit from increased communication with healthcare professionals. Therefore, support programmes should include all close FC, which is more common in existing evaluated psychosocial interventions for caregivers in the HSCT context [42] compared to general cancer care, where generally only partners are included [43]. Reported support needs among FC in the present study are associated with worse caregiver outcomes. Such correlations are in line with earlier studies, both in the auto- and allo-HSCT context [15] and in the palliative context [18, 19]. In particular, the two top concerns are associated with higher caregiver burden, symptoms of anxiety and depression and lower preparedness for caregiving prior to allo-HSCT. A larger review proves that FC in HSCT often do whatever it takes to get through the situation [4], but that this has a price, as it frequently results in decreased quality of life even in the survivorship phase [4, 44, 45]. In the present study, having time for oneself and receiving practical help were associated with general health, indicating that some FC do not manage to balance the demands with their own capacity [4]. In addition, it should be remembered that earlier studies on couples in cancer care reported that patients and partners react as an ‘emotional system’ meaning that if the psychological needs of FC are not addressed, it has a great influence on the patient’s well-being [11].

In summary, the FC in this study reported many different support needs, and the most prominent support needs were to handle the uncertainty and their own worries throughout the allo-HSCT trajectory, especially younger FC and those who were not a partner. One possible way to help these FC could be to use the CSNAT to identify the individual needs. In our earlier interview study, we noted that the FC were so preoccupied with the recipients’ health and well-being that some were unable to focus on themselves [13]. This result together with the findings from the present study implies that FC needs help to address their support needs. One way to do this is to use the CSNAT tool, which provides a structure for dialogue that enables focus on the dual roles of FC, caring for the recipient as well as for themselves, which is also suggested by the constructors of the original too [46]. Using the CSNAT tool repeatedly throughout an allo-HSCT trajectory and delivering individualized support interventions to FC may decrease their caregiver burden over time, as shown in palliative care [38]. An intervention study is needed to evaluate the usability and effects of employing the CSNAT in the clinical context of FC of allo-HSCT recipients.

One important limitation in the present study is the small sample size, explained by the fact that allo-HSCT recipients constitute a relatively small population. If FC from all six centres in Sweden had been included, it would have increased the sample size; however, this was not possible due to logistical and organizational reasons. Simultaneously, these FC are in a vulnerable situation, which is reflected in the high support needs reported in our study. Nevertheless, the response rate was high (70%). However, in an earlier qualitative study [13], our research team showed that it might be difficult to get FC with higher support needs to participate in research studies. Based on these findings, it cannot be excluded that FC who agreed to participate had lower support needs than the dropouts and those who declined. Thus, the support needs in the population is probably higher than reported in the present study. The CSNAT is designed to be used as a tool in an assessment conversation between FC and staff in clinical practice; however, in this exploratory study, it was used as a survey on four occasions to achieve a picture of FC needs over time in a new context, the allo-HSCT trajectory. Therefore, a limitation might be that the answers reflect the FC spontaneous understandings of the questions, without any possible conversations with clinical staff. However, the CSNAT has been used earlier as a survey in validation studies [18, 19] and to evaluate a care model [47].

A strength of our study is the use of a study coordinator who phoned all participants who did not return their questionnaire in time to remind them, which probably increased our response rate in the three follow-ups. These phone calls also provided information showing that both those FC who had a tough time as well as those who thought everything was fine dropped out, especially in the last follow-up. One problem with the small sample is that the statistical power is somewhat low with increased risk of type II errors. This risk is largest in relation to the repeated measures, because due to attrition, only 50 FC participated in the final follow-up. For this reason, no correction for the multiple tests in the post hoc analysis was used. Recommended methods for handling this problem, such as Bonferroni corrected *p* values, were deemed too conservative in the present study.

## Conclusions

This rather small study reports that support needs among family caregivers are associated with worse caregiver outcome prior to allo-HSCT and further that most of their support needs do not diminish over time from before until 4 months after. This indicates that family caregivers need more attention during an allo-HSCT.
